# Identification of nodes influence based on global structure model in complex networks

**DOI:** 10.1038/s41598-021-84684-x

**Published:** 2021-03-17

**Authors:** Aman Ullah, Bin Wang, JinFang Sheng, Jun Long, Nasrullah Khan, ZeJun Sun

**Affiliations:** 1 School of Computer Science and Engineering, Central South University, Changsha, 410083 China; 2 Big Data Institute, Central South University, Changsha, 410083 China; 3 College of Computer Science and Technology, Nanjing University of Aeronautics and Astronautics, Nanjing, 210016 China; 4 Department of Computer Science, COMSATS University Islamabad, Vehari Campus, Vehari, 61100 Pakistan; 5 School of Information Engineering, Pingdingshan University, Pingdingshan, Henan China

**Keywords:** Computer science, Information technology

## Abstract

Identification of Influential nodes in complex networks is challenging due to the largely scaled data and network sizes, and frequently changing behaviors of the current topologies. Various application scenarios like disease transmission and immunization, software virus infection and disinfection, increased product exposure and rumor suppression, etc., are applicable domains in the corresponding networks where identification of influential nodes is crucial. Though a lot of approaches are proposed to address the challenges, most of the relevant research concentrates only on single and limited aspects of the problem. Therefore, we propose Global Structure Model (GSM) for influential nodes identification that considers self-influence as well as emphasizes on global influence of the node in the network. We applied GSM and utilized Susceptible Infected Recovered model to evaluate its efficiency. Moreover, various standard algorithms such as Betweenness Centrality, Profit Leader, H-Index, Closeness Centrality, Hyperlink Induced Topic Search, Improved K-shell Hybrid, Density Centrality, Extended Cluster Coefficient Ranking Measure, and Gravity Index Centrality are employed as baseline benchmarks to evaluate the performance of GSM. Similarly, we used seven real-world and two synthetic multi-typed complex networks along-with different well-known datasets for experiments. Results analysis indicates that GSM outperformed the baseline algorithms in identification of influential node(s).

The concepts of complex networks are abstracted from real-world system networks^1–3^, like transport system networks, human friendship, web hyperlink and protein networks, etc. Such networks have common characteristics, i.e., heterogeneous topologies, that make it impossible to assign the same importance to each node in the network. Therefore, it is essential to identify the influential nodes via quantitative approaches to examine their properties and to practice their proper usage. For example, in infectious diseases network, preventing the spread of rumors and viruses^4,5^, in criminal networks, quickly identifying a terrorist organization’s leader^6^. Similarly, in the traffic system network^7^, food chain network^8^, drug network^9^, and so on. There are a good number of studies have been proposed and deployed in the field of complex network on the identification of influential nodes^10–12,12–20^ where identification of most important nodes from local and global perspectives is worth mentioning^21^. Although closeness centrality (CC) and betweenness centrality (BC)^22^ are path-based indicators that consider the global structure of the network to identify the influence of nodes. However, due to their high computational complexity, they may not be applicable to many networks. It’s common that degree centrality (DC)^23^ is the simplest method to identify the influential nodes, but it fails to consider the global structure of networks. Kitsak et al.^24^ proposed the k-shell method for locating influential nodes, but it is too coarse to identify the required influential nodes. Besides these, iterative-based centralities such as eigenvector centrality (EC)^25^, page rank (PR)^26^, hypertext induced topic search (HITS)^27^, and son on are not appropriate for networks with tight connections.

Recently, some approaches have put efforts forward for the identification of influential nodes such as profit leader^28^, inverse square law^29^ and gravity index model^30^. The profit leader method considers the set of important nodes on the basis of the profit leader concept analysis. In the paper by L.-l. Ma et al.^30^ proposed gravity formula-based algorithm, which considers both neighbour’s nodes influences and path information. However, in some cases, it is also important to combine the global as well as local structure of nodes in the entire network. Similarly, the inverse square model is based on node interactions and is not suitable for large network. Furthermore, in view of propagation probability, Ma et al.^31^ proposed hybrid degree centrality (HC), which combines local indicators and degree centrality measures. All in all, these approaches have their own shortcomings and limitations. Still, the identification of influential nodes is a challenge.

From the above discussion, to address these changeling problems, inspired from literature^10,29,30^, in this study, we design a new mechanism called GSM that not only considers the self-influence of the node in the network but also concentrates on the global influence of nodes. To analyze the algorithmic performance, we employed GSM on different kinds of real as well as synthetic networks where we used the susceptible-infected-recovered (SIR) and kendall’s $\tau$ coefficient models to examine the effectiveness of GSM. In addition, we compared the experimental results of the baseline algorithms and with recently proposed approaches, where simulation results on seven different types of real and two synthetic networks showed that GSM effectively identifies influential nodes.

The framework of the paper is organized as follows: We present preliminaries and a brief introduction of baseline algorithms, including BC, PL, GIC, HI, CC, ECRM, DNC, IKH and HITS in Preliminaries section. The proposed GSM model is presented in Proposed method. Results and discussion to illustrate the effectiveness of the GSM are discussed in Results and discussion section, and finally, some conclusion and future recommendations are given in Conclusion and future recommendations section.

## Preliminaries

A network can be denoted by G, equated as G = (V, E), where V and E represent nodes and edges, respectively. Moreover, Betweenness Centrality (BC)^32^, Closeness Centrality (CC)^33^, HITS^34^, H-Index (HI)^35^, Profit Leader (PL)^28^, Improved k-shell hybrid method (IKSh)^12^, Extended Cluster Coefficient Ranking Measure (ECRM)^36^, Gravity Index Centrality (GIC)^30^ and Density centrality (DNC)^37^ are introduced in this section.

1. Betweenness Centrality (BC): BC calculates influential nodes based on global information^32^. $BC_(i)$ is defined as:
  1 $$\begin{aligned} BC_(i)=\sum _{j,k\ne 1} \frac{g_{jk}(i)}{g_{jk}}, \end{aligned}$$
  where $g_{jk}$ indicates number of paths between nodes *j* and *k*, and $g_{jk}(i)$ represents the shortest paths between nodes *j* and *k*, that pass through node *i*.
2. Closeness Centrality (CC): CC also calculates influential nodes based on global information. It uses the shortest distance between each pair of nodes to identify the influence of each node^33^. CC of node *i* is defined as:
  2 $$\begin{aligned} CC_(i)=\frac{N-1}{\sum _{j\ne 1} d_{ij}}, \end{aligned}$$
3. Hyperlink Induced Topic Search (HITS): This algorithm is based on two factors i-e., Authority Update, and Hub Update. Authority update is computed by considering the number of hub edges associated with the authority website, and Hub Update is computed by considering the number of authority websites linked by the Hub website^34^.
4. H-Index (HI): This algorithm identifies the influential node’s by taking into account the node’s neighbor and using H-index notation. A high H-index represents that the node has more important than other connected nodes^35^.
5. Profit Leader (PL): This algorithm is based on profit leader concept analysis and suitable for any network i.e., directed or undirected^28^.
6. Improved K-shell Hybrid (IKH): This algorithm considers the k-shell, shortest distance between the nodes and parameter $\lambda$ (in range between 0 and 1) to identify the most influential nodes^12^.
7. Gravity Index Centrality (GIC): This algorithm basis on universal gravity concept; that considers both neighbor’s nodes influences and path information^30^. GIC(i) is defined as:
  3 $$\begin{aligned} GIC(i)= \sum _{j\epsilon \theta _i}\frac{kshell(i)\times kshell(j)}{dist_{i,j}^2}, \end{aligned}$$
  where $\theta _i$ is the set of neighbors node *i*.
8. Extended Cluster Coefficient Ranking Measure (ECRM): This algorithm is working on the basis of local clustering coefficients and uses link similarity between adjacent nodes^36^.
9. Density centrality (DNC): It is inspired by the area density formula to identify the influence of nodes in the spreading dynamics^37^. DNC(i) is defined as:
  4 $$\begin{aligned} DNC(i)= \sum _{j\epsilon \xi _i}\frac{degree_i}{\pi d_{i,j}^2}, \end{aligned}$$

## Proposed method

Several approaches based on the global structure of the network to identify the influence of nodes have been developed and deployed, but better utilization of self as well as global structure influence is still a challenge, needs to be addressed. Inspired from literature^37–39^ , a Global Structure Model (GSM) was proposed, which consists of self and global influences.

### Self-influence

In this context, we used e (natural logarithm) and take k-shell $Ks(v_i)$, and nodes number (N) in the network as power parameters to minimize the overestimation of the self-influence.

5 $$\begin{aligned} SI(v_i)= e^{\frac{Ks(v_i)}{N}} \end{aligned}$$

where N shows the number of all nodes in the network.

### Global-influence

The node influence also considers the influence of the other connected nodes to it. Normally, the node influence is increased if its neighborhoods have a high value of k-shell; however, the contact distance between the two nodes cannot be ignored, which is inversely proportional to the influence of the nodes.

6 $$\begin{aligned} GI(v_i)=\sum _{i\ne j}{\frac{Ks(v_j)}{d_{ij}}} \end{aligned}$$

where $d_{ij}$ is the shortest distance between node *i* and node *j*.

### Node influence

The node $V_i$ influence is not only on its own influence but also on the nodes around it. Therefore, the proposed GSM simultaneously considers these two aspects, self and global influence, which can be defined as,

7 $$\begin{aligned} GSM(v_i)=SI(v_i)\times GI(v_i) \end{aligned}$$

We can also express GSM of the node $v_i$,

8 $$\begin{aligned} GSM(v_i)=e^{\frac{Ks(v_i)}{N}}\times \sum _{i\ne j}{\frac{Ks(v_j)}{d_{ij}}} \end{aligned}$$

where ${Ks(v_i)}$ and ${Ks(v_j)}$ denote the k-shell of node *i* and node *j*,

### Computation process

**Figure 1:**
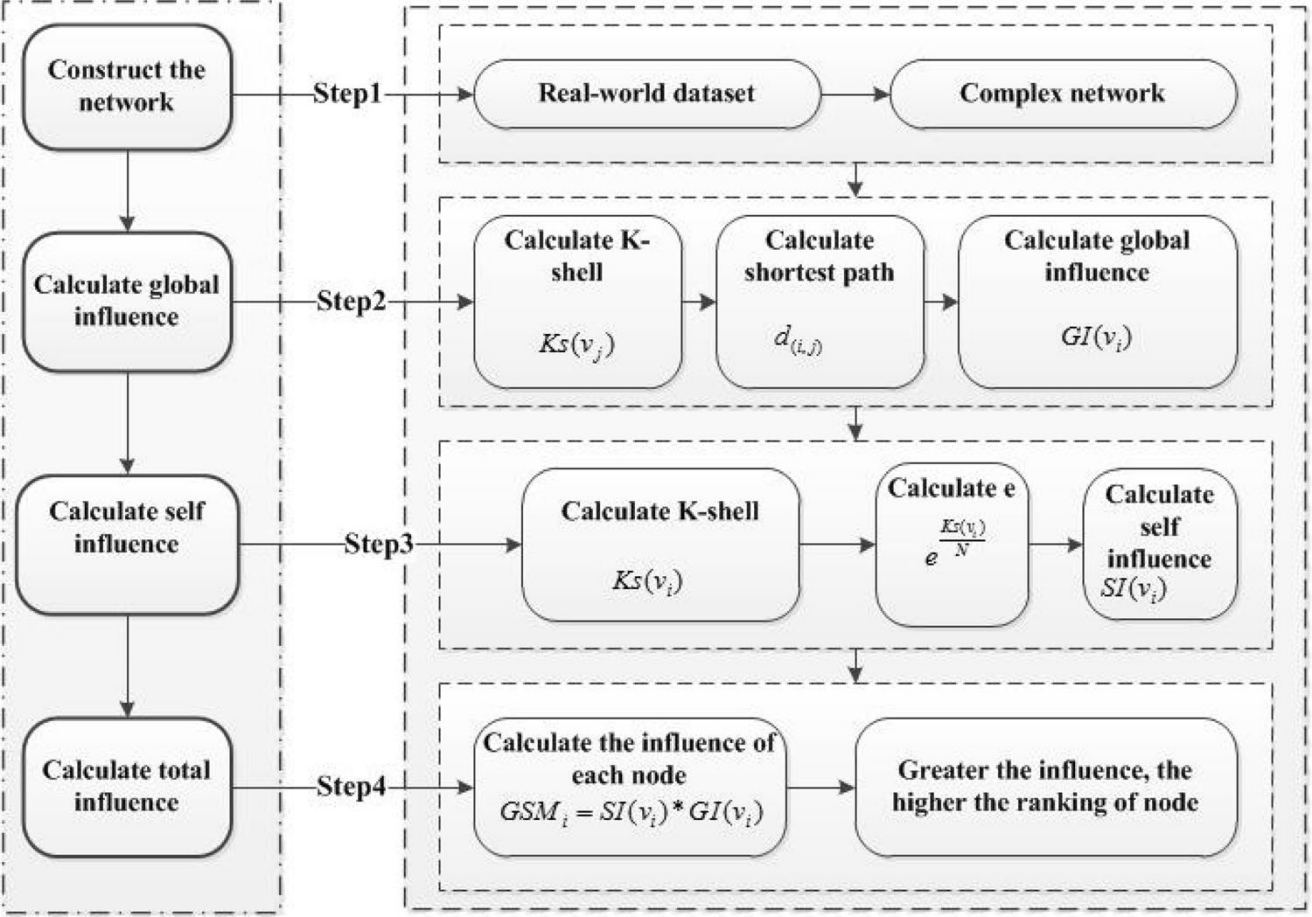
The flow chart of the proposed GSM.

**Figure 2:**
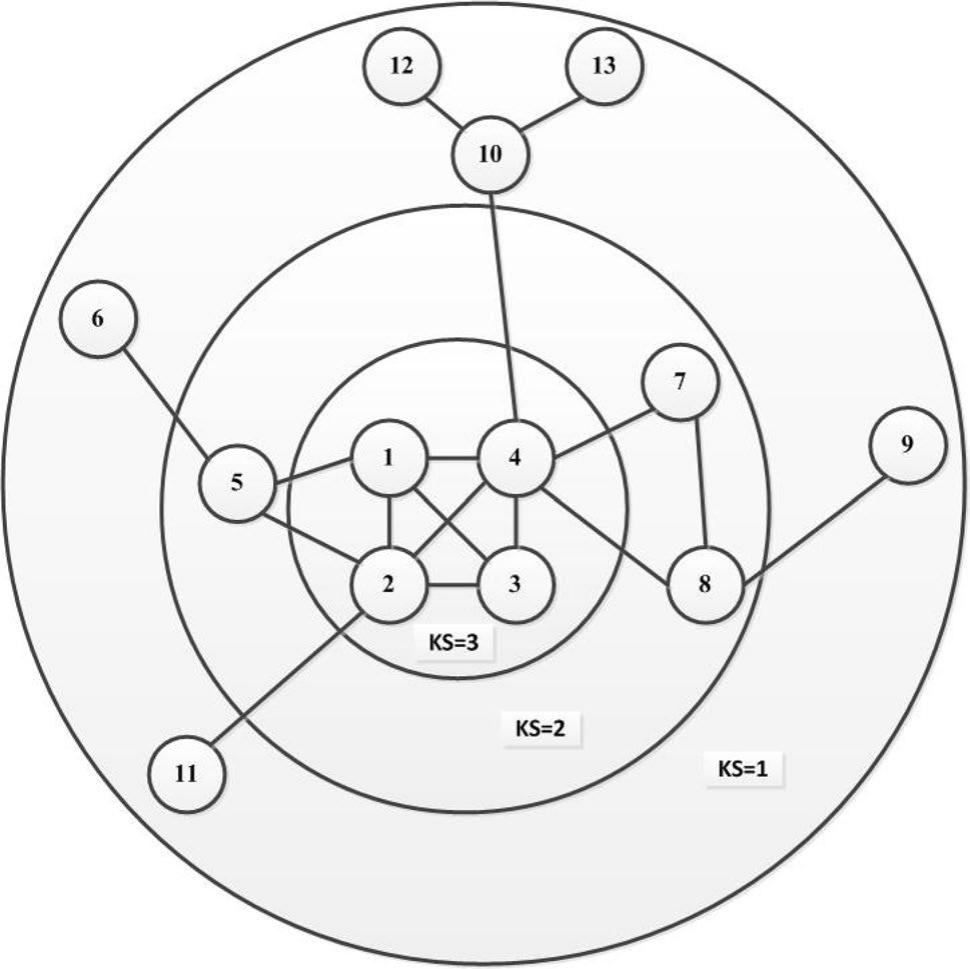
A network with 17 edges and 13 nodes.

The proposed GSM model is divided into four parts; first, construction of corresponding network; second, calculation of the network’s global influence and the k-shell of node and the distance between nodes. In the third step, we consider the self-influence of the network, the self influence of the node itself is computed. Finally, the influence of each node on the entire network is calculated. To further demonstrate GSM method, as shown in Fig. 1, for a specific calculation process, here we consider a simple network to clarify it in detail. In Fig. 2, consists of 13 nodes and 17 edges. As shown in the network, we consider GSM method by taking the node V4 influence as an example. First, we calculate the k-shell and the shortest distance between each node; we have

$$KS1=3, KS2=3, KS3=3 KS4=3, KS5=2, Ks6= 1, KS7=2, KS8=2, KS9=1, KS10=1, KS11=1, KS12=1, KS13=1,$$ d4-1 = 1 d4-2 = 1,d4-3 = 1, d4-5 = 2, d4-6 = 3, d4-7 = 1, d4-8 = 1, d4-9 = 2, d4-10 = 1, d4-11 = 2, d4-12 = 2, d4-13 = 2.

To calculate the self influence and global influence, here we apply Eqs. (6) and (7); we have, $S(4)=e^{\frac{3}{13}}=1.25956$, and $GI(4-1)={\frac{3}{1}}=3$ $GI(4-2)=3$, $GI(4-3)=3$, $GI(4-5)=1$, $GI(4-6)=0.3333$, $GI(4-7)=2$, $GI(4-8)=2$, $GI(4-9)=0.5$, $GI(4-10)=1$, $GI(4-11)=0.5$, $GI(4-12)=0.5$, $GI(4-13)=0.5$, we have added all these values based on Eq. (7), we have, $GI(4-1)+GI(4-2)+GI(4-3)+GI(4-5)+GI(4-6)+G(4-7)+GI(4-8)+GI(4-9)+GI(4-10)+GI(4-11)+GI(4-12)+GI(4-13)=17.333333$, Finally, the influence of node V4 can be calculated, we have $GSM_4= 1.25956 \times 17.333333= 21.833$. Table 1 shows the ranking influence of each node in the given simple network.

## Experiments

Experimental setup is performed on system with configuration as: CPU: Feiteng 1500A (1, 16-core, 1.5 ghz), Operating system: galaxy kirin server os Bios: China-made Kunlun firmware Memory: 3 GB and Hard disk: 2 TB (Fig. 2).

### Evaluation metrics

#### SIR model

We used the SIR model to investigate the spreading dynamic of each node^40,41^ to quantify the performance of GSM and other benchmark centralities. In the SIR model, there are three states, (i) Susceptible (S), (ii) Infected (I), (iii) Recovered (R). Susceptible (S) refers to a healthy state and can be infected by others. Infected (I) refers infected state and can infect other individuals. Recovered (R) denotes a recovered state, which cannot be infected by other individuals again. For the first time, all the seed nodes are in a susceptible form. At each time step, the seed node can infect its nearest and next-nearest neighbor nodes (in the susceptible state) with a probability $\beta$, then each node (the node which was infected) enters into the recovered state with a probability $\mu$. This process continued till there are no more infected nodes. Finally, all the recovered nodes are used to simulate the actual node impact. Here, S(t), I(t), and R(t) indicate the nodes numbers in susceptible, infected, and recovered states, respectively. Therefore,

9 $$\begin{aligned} \left\{ \begin{array}{lr} \frac{ds(t)}{d(t)} = -\beta s(t)i(t), &{} \\ \frac{di(t)}{d(t)} = \beta s(t)i(t) - \beta i(t), &{} \\ \frac{dr(t)}{d(t)} = \beta i(t). &{} \end{array} \right. \end{aligned}$$

The spreading influence $K_i$ of the node $V_i$ follows as

10 $$\begin{aligned} K_i=F(t)=\frac{1}{N_{ite}}(nI+nR) \end{aligned}$$

where $N_{ite}$ indicates iteration numbers, *nI* and *nR* are the number of infected and recovered nodes, respectively. Where we set $N_{ite}$= 1000 independent run.

**Table 1:** GSM results of Figure 2.

Node	4	2	1	3	5	8	7	10	11	9	6	12	13
Influence	21.833	20.15	19.52	18.053	15.06	15.06	14.48	14.12	14.12	10.02	9.91	9.21	9.21

#### Kendall’s Tau $(\tau )$

We used kendall’s $(\tau )$^42,43^ to calculate the performance of GSM further. Let suppose, two-node sequences $(X \& Y)$ are correlated with similar nodes number (n), $X=(x_1,x_2,\ldots ,x_n)$ and $Y=(y_1,y_2,\ldots ,yn)$. One pair of two annotations $(x_i, y_i)$ and $(x_j, y_j)$ $(i\ne j)$ are said to be concordant if the ranking of both component agree, i-e. if both $xi > xj$ and $y_i > y_j$ or $x_i < x_j$ and $y_i < y_j$. They are said to be discordant if $x_i > x_j$ and $y_i < y_j$ or $xi < x_j$ and $y_i > y_j$ or if $x_i = x_j$ or $y_i = y_j$ , the pair is neither concordant nor discordant. The kendall’s $(\tau )$ is defined as:

11 $$\begin{aligned} \tau (X,Y)=\frac{n_{c}-n_{d}}{n(n-1)/2} \end{aligned}$$

where $n_c$, and $n_d$ denote the number of concordant and discordant pairs, respectively.

### Datasets description

#### Real-world networks

We evaluated GSM on seven different real-world networks to validate its efficiency. The seven real networks are publicly available and can be obtained from (http://networkrepository.com). The datasets are, e.g., (i) Jazz^44^: this is a communication network , consist of 198 nodes and 2472 edges. (ii) H-friendship network^45^: this network reflects user friendship and contains 12534 edges and 1858 nodes. (iii) E-mail network^46^: this is a communication network of the Roviraa Virgilli University of Spain, where nodes are e-mail user, and edges represent at least one e-mail was sent. (iv) Crime network^47^: this network consists of 829 nodes and 1476 edges . (v) Dolphin^48^: it is a social network with 62 nodes and 159 edges. (vi) Web-spam^49^: this is a famous social netwok provided by Purdue University, which consists of 4767 nodes and 37375 edges. (vii) Astroph-e^50^: this network consists of 18771 nodes and 198,050 edges. The topological characteristics of the seven real-world multi-typed complex networks are shown in Table 2.

#### Synthetic networks

There is a bulk of exemplary complex networks exist in the real world and we do not know the details of ground realities about all of them because it’s not even possible to conceal such a large number of information about a matter that is so widely being exploited. Therefore in order to evaluate GSM and the baseline methodologies, we applied the benchmark generator model^51,52^ to generate different synthetic networks for the process of experimentations. (i) Random network: it is an synthetic random network in which we set the number of nodes as V= 1000, probability 0.01, the average degree as $<k>= 3$, and it consists of E = 4852 edges. (ii) BA network: this synthetic network consists of V = 2000 nodes and E = 7984 edges and 4 edges added are each time.

**Table 2:** The topological characteristics of the seven different real networks, where E and V are the numbers of edges and nodes.

Network	${\hbox {|E|}}$	${\hbox {|V|}}$	${{\hbox {d}}}_{avg}$	${{\hbox {d}}}_{max}$	${\hbox {<CC>}}$
H-friendship	12534	1858	13.49	272	0.141
Jazz	2472	198	27	100	0.6174
E-mail	5451	1133	9	71	0.2202
Crime	1476	829	3	25	0.0058
Dolphin	159	62	5	12	0.2589
Web-spam	37,375	4767	15	477	0.2859
Astroph-e	198,050	18,771	21	504	0.6306

The $d_{avg}$ , $d_{max}$ and $<CC>$ represents the maximum degree, averages degree, and average cluster coefficient of each network.

## Results and discussion

To measure the influence of nodes in different real and synthetic networks and to validate the applicability and effectiveness of the GSM, we used two evaluation metrics i-e., SIR, and Kendall’s models. First, we used a simple graph containing 13 nodes and 17 edges, as shown in Fig. 2, applied GSM to find the influential nodes and, results are compared and analyzed with the outcomes of the rest of the benchmark algorithms such as BC, CC, HITS, HI, GIC, DNC, IKH, ECRM and PL.

**Figure 3:**
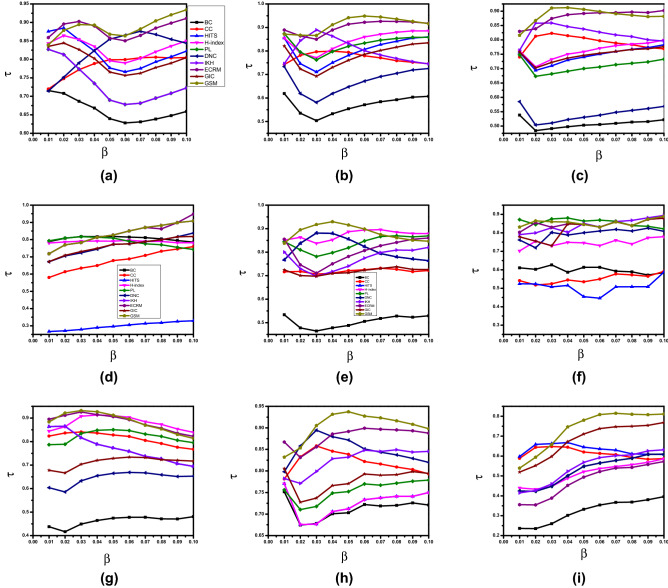
Kendall coloration coefficient $\tau$ results are obtained by comparing the ranking results generated by 10 algorithms and the SIR model. The infection probability $\beta$ of the network is set in the range between range between 0.01 to 0.10, and results are calculated based on average outcome of 1000 independent runs. Where (**a**) represents E-mail, (**b**) H-friendship, (**c**) Web-spam, (**d**) Crime, (**e**) Jazz (**f**) Dolphin, (**g**) Astroph-e, (**h**) Random, (**i**) BA, etc.

Kendall’s $(\tau )$ of the proposed GSM and other algorithms are shown in Fig. 3. As, it can be seen that in terms Kendall $(\tau )$, GSM achieves higher values, i.e., the values in range from 0.9 to 1 for $\beta =0.01-0.1$, shows that GSM is performance-wise better on different networks, such as Dolphin, H-friendships, Jazz, Crime, E-mail, Web-spam, Astroph-e, BA, and Random networks.

**Table 3:** Top-10 ranking nodes of the Dolphin network using ten different methods.

Rank	BC	CC	HITS	HI	PL	DNC	IKH	ECRM	GIC	GSM
1	37	37	15	15	15	15	15	15	15	38
2	2	41	38	17	46	38	38	38	38	15
3	41	38	46	19	38	46	46	46	46	46
4	38	21	34	21	34	34	21	21	21	21
5	8	15	51	22	52	52	34	34	41	34
6	18	2	30	25	58	21	41	30	34	51
7	21	8	52	30	21	30	37	41	37	41
8	55	29	17	34	14	41	30	52	51	30
9	52	34	41	38	30	18	52	51	30	37
10	58	9	22	41	18	58	51	39	2	52

**Table 4:** Top-10 ranking nodes of the Crime network using ten different methods.

Rank	BC	CC	HITS	HI	PL	DNC	IKH	ECRM	GIC	GSM
1	815	815	425	95	110	815	815	2	815	815
2	2	110	110	110	95	425	2	815	110	110
3	110	2	95	39	2	110	110	425	425	425
4	56	56	715	2	425	56	425	110	56	56
5	356	425	2	10	815	220	56	56	220	404
6	425	404	56	356	56	2	220	220	404	220
7	220	46	695	404	404	356	356	356	356	356
8	39	39	59	46	220	153	404	43	39	695
9	43	43	531	51	356	43	39	39	95	95
10	14	356	43	56	715	514	43	514	514	514

**Figure 4:**
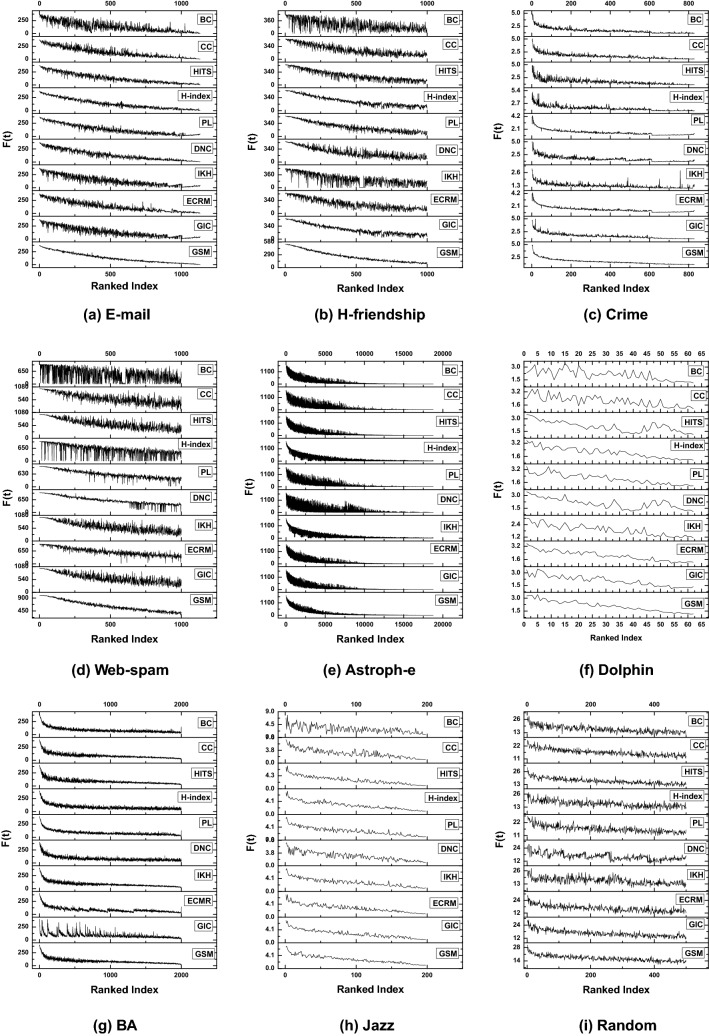
Propagation influence of ranking effect using GSM and other baseline benchmarks, where F(t) represents the number of infected and recovered nodes at the time (t), and horizontal index line means the ranking order list. For big networks we set $\lambda = 0.01$ such as (Astroph-e, Web-spam, BA and H-friendship) networks and for small networks (Dolphin, Jazz, Crime, Random, and E-mail), we set $\lambda = 0.10$.

In order to further examine the propagation effect of GSM, we analyzed the spreading impact of the ranked nodes in the SIR model. To better distinguish the influential nodes, the infection probability $\lambda$ needed to be set in the range between 0.01 and 0.1. For big networks (Astroph-e, Web-spam, BA and H-friendship), we set $\lambda = 0.01$ because, in case of bigger values, propagation will occur across the whole network^24^. Where it is not easy to differentiate the importance of distinct nodes. For small networks (Dolphin, Jazz, Crime, Random, and E-mail), we set $\lambda =0.1$, and also we set the recovery probability $\epsilon = 1$ and the time t = 1000. First, the influence of each node is computed using different algorithms, and then sorted in descending order. Tables 3 and 4 shows the top ten ranked nodes; due to the limited space, only we present the top ten nodes of two networks Dolphin and Crime. We observed that most of the top-10 nodes of our algorithm are also exist in other algorithms. Hence, the proposed GSM validity is verified. Second, each ranked node is treated as a seed node to impacting other ranked nodes. Finally, we computed the infected numbers of nodes for each seed node through an average of over 1000 turns. Figure 4 indicates the results of the average infected number of nodes using ten algorithms. In general, more influential nodes can infect more nodes, so an efficient and effective method can create a curve that decreases from left to right. As shown in Fig. 4, our proposed GSM gets a better infection effect than other methods on different networks.

**Figure 5:**
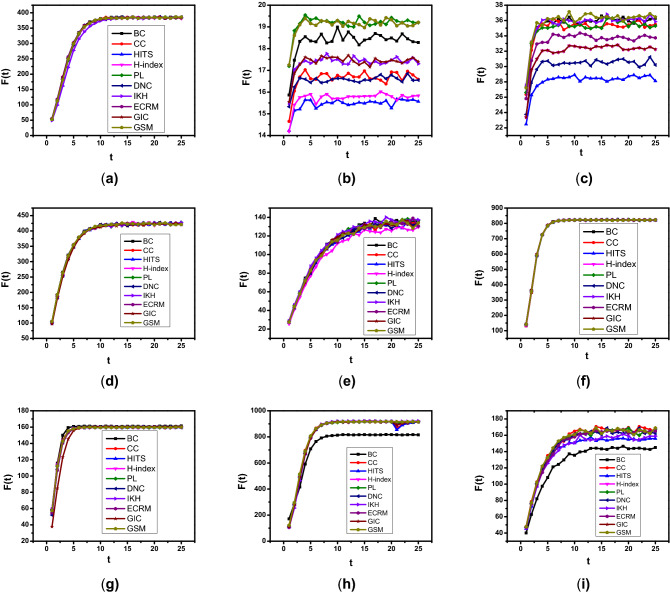
Propagation influence of top-10 ranking effect using GSM and the corresponding baseline measures, where F(t) represents the number of infected and recovered nodes at time (t), and horizontal index line represents the ranking order list. Where (**a**) represents E-mail, (**b**) Dolphin, (**c**) Crime, (**d**) BA, (**e**) Random (**f**) H-friendship, (**g**) Jazz, (**h**) Web-spam, (**i**) Astroph-e, etc.

Moreover, we compared the top ten nodes’ effects selected by our proposed GSM and the corresponding baseline centrality measures for different networks. All top ten nodes are considered as seed nodes and the time t in the range between 1 and 25. Figure 5 illustrates the influence of the top ten nodes in nine different networks; as can be seen, the proposed GSM gets the highest spreading efficiency than other centralities. In addition, It clearly shows that when the infection F(t) increases as time t increases and finally gets a steady value at a time t after consecutive time point. Since there are ten seed nodes, and most network propagation arrives in a steady state on time t=25, where we analyzed the spreading effects of GSM and the rest of other centralities measures.

## Computational complexity of GSM

There are two main components of the proposed GSM. In the first stage, the time complexity of the node’s global influence is calculated. We used Dijkstra to calculate the shortest distance, and its complexity is $O(n^2)$. In the second stage, the time of complexity is *O*(*n*). Therefore, the total computational complexity of GSM is $O(n^2)$. Table 5 lists the computational complexity of the proposed GSM and other benchmarks, as we can see that the computational complexity of GSM is not very low, but its accuracy is better than other benchmarks, and also GSM can automatically measure nodes influence without any parameters (shown in Figs. 3, 4 and 5). In future work, we plan to enhance GSM as paralleling computations.

**Table 5:** Computational complexity of GSM and other benchmarks.

Methods	Complexity
BC	$O(n^2m)$
CC	*O*(*nm*)
HITS	*O*(*n*)
HI	*O*(*nlogn*)
PL	$O(n < k >)$
DNC	$O(n^3)$
IKH	$O(n^*<k>^r)$
ECRM	$O(|e| + |n|)$
GIC	$O(n^2)$
GSM	$O(n^2)$

## Conclusion and Future Recommendations

We studied the problem of identification of nodes influence in complex networks. Several approaches have been developed and deployed in this area but still, it is a big issue for scientists and researchers. In this regard, we proposed an algorithm called GSM to identify influential nodes, which considers both self as well as global influence of nodes in the networks. We applied the proposed GSM on different real as well as synthetic networks and employed two evaluation metrics (SIR and Kendall $\tau$) to verify its efficiency. Experimental results demonstrated that our algorithm performed better than the benchmarks. For further work, the proposed GSM algorithm can be extended to many forms for better results. For instance, adding some parameters to control the intensity among various nodes to yield better performance. Furthermore, we also plan to combine the profit leader algorithm concept with the proposed algorithm to enhance the performance.

## Data Availability

All the real networks are available publicly and can be accessed from http://networkrepository.com.
